# Influence of Annealing Temperature on Optical Properties of Sandwiched ZnO/Metal/ZnO Transparent Conductive Thin Films

**DOI:** 10.3390/mi13020296

**Published:** 2022-02-13

**Authors:** Qijing Lin, Fuzheng Zhang, Na Zhao, Ping Yang

**Affiliations:** 1 Collaborative Innovation Center of High-End Manufacturing Equipment, Xi’an Jiaotong University, Xi’an 710054, China; xjjingmi@163.com; 2 State Key Laboratory of Mechanical Manufacturing Systems Engineering, Xi’an Jiaotong University, Xi’an 710049, China; xjzfz123@stu.xjtu.edu.cn (F.Z.); ipe@xjtu.edu.cn (P.Y.); 3 School of Mechanical and Manufacturing Engineering, Xiamen Institute of Technology, Xiamen 361021, China

**Keywords:** optical properties, sheet resistance, transparent conductive thin film, annealing temperature, ZnO/Metal/ZnO

## Abstract

Two sandwiched ZnO/Metal/ZnO transparent conductive thin films, 50nm ZnO/Cu/50nm ZnO (abbreviated as ZnO(Cu)) and 50nm ZnO/Ti/Cu/Ti/50nm ZnO (abbreviated as ZnO(Ti/Cu)) were deposited by magnetron sputtering technology. The comparative analysis of experiment results shows that the introduction of the Ti layer is beneficial to the overall properties of ZnO(Ti/Cu) thin film compared to ZnO(Cu) thin film with the same metal layer thickness. The effect of the annealing temperature on the performance of the two film systems was studied. Although the carrier concentration did not always increase with annealing temperature, the sheet resistances did decrease due to the obvious increase of mobility. The transmittance of ZnO(Cu) thin films increases with annealing temperature, while that of ZnO(Ti/Cu) films increases at first and then decreases. The optical band gap of ZnO(Cu) thin films increases with temperature, but is lower than that of ZnO(Ti/Cu) thin films, whose bandgap first increases with temperature and then decreases. The figure of merit of the ZnO(Ti/Cu) film is better than that of ZnO(Cu), which shows that the overall performance of ZnO(Ti/Cu) films is better, and annealing can improve the performance of the film systems.

## 1. Introduction

As a transparent conductive oxide, zinc oxide (ZnO) is a wide-bandgap semiconductor with high transmittance in the visible range [1,2], which has led to its broad application in the fields of flat panel displays, camera tubes, solar cells, organic light-emitting diodes, liquid crystal displays and so on [3,4]. However, pure ZnO has a high resistivity and cannot meet the requirements of transparent conductive films due to two mutually restrictive factors: high transmittance and low resistivity in the visible light range. Sandwiched ZnO/Metal/ZnO multilayer thin film combining metal with a semiconductor can effectively improve the electrical conductivity without reducing the optical transmittance [5] because the metal in the middle layer can conduct electrons in order to reduce the resistivity. Various metal-sandwich ZnO multilayers, such as ZnO/Ag/ZnO [6], ZnO/Au/ZnO [7], ZnO/Al/ZnO [8] and ZnO/Cu/ZnO [9,10,11,12], have been developed. Due to the positive effect of copper in improving electrical conductivity and optical properties, ZnO(Cu) sandwiched multilayer has attracted much interest.

Various technologies, such as magnetron sputtering [11,12], atomic layer deposition [13] and sol-gel [14], have been used to fabricated ZnO(Cu) sandwiched films on different substrates, such as glass [11,12,13,14] and flexible polyethylene naphthalate [15]. Simulation and experimental methods were used to study the influence of Cu and ZnO layer thickness on the performance of ZnO(Cu) sandwiched film. Both theoretical analysis and experimental results prove that the electrical conductivity and optical properties of oxide transparent conductive films with a Cu interlayer depend considerably on its thickness [10,11,12,13,14,15,16,17,18]. Generally speaking, electrical conductivity will be improved with the increase of Cu layer thickness because of its excellent conductivity, but the transmittance will increase at first, reach the maximum value at a certain thickness, and then decrease sharply because of the absorption in the Cu layer. Although ZnO is one of the best choices for the semiconductor layer, oxygen chemisorption on its surface and grain boundaries of ZnO result in higher resistivity [17]. Therefore, the electrical properties of pure ZnO are unsuitable. To solve this problem, ZnO films are usually treated by annealing to improve the stability of the film by releasing strain energy and improving the crystal shape [19]. Previous studies [16,18] have shown that the annealing atmosphere and temperature affect the properties of the ZnO/Cu/ZnO multilayers.

Reducing the absorption of light in the metal layer is an effective measure to improve the properties of metal-sandwich ZnO multilayers. However, ultrathin Cu films (i.e., less than 10 nm) are susceptible to oxidation and corrosion, which significantly affect their electrical and optical properties [15]. One solution is to cover the layer with a protective, ultrathin film with stronger reducibility. Previous research has demonstrated that a continuous, ultrathin Cu/Ti bilayer film in which the Ti film acts as a protective film can improve the performance and stability of the transparent conductive electrode [19,20,21].

In this paper, two ZnO-based transparent conductive thin-film systems, consisting of either sandwiched Cu or Ti/Cu/Ti metal layers, were fabricated using magnetron sputtering technology and were annealed at temperatures from 100 °C to 400 °C in an Ar atmosphere.

Comparative experiments and characterization analysis, such as microstructure analysis, sheet resistance and optical properties, were investigated, and the influence of annealing temperature on performance was studied.

## 2. Experiment

Two ZnO transparent conductive thin-film systems (50nm ZnO/Cu/50nm ZnO and 50nm ZnO/Ti/Cu/Ti/50nm ZnO) were fabricated on glass substrates using magnetron sputtering technology. The sputtering equipment (Explorer14, Seattle, WA, USA) had three target guns, allowing it to perform RF sputtering of ZnO and the DC sputtering of Cu and Ti (both purity grades were 99.99%) in an Ar atmosphere with a base pressure of 2 × 10^−5^ Pa. Then, the fabricated thin-film systems were annealed in a high temperature furnace for 30 min at temperatures from 100 °C to 400 °C in an Ar atmosphere.

The crystallization structures of the two ZnO transparent conductive thin-film systems created at the various annealing temperatures were determined using X-ray diffraction (XRD) (Xpert Pro, Rotterdam, The Netherlands). In order to evaluate the performance of the two film systems, the four-point probe method and ultraviolet and visible spectrophotometer (Shimadzu UV-3600, Kyoto, Japan) were used to measure the sheet resistance and transmission spectra, respectively.

## 3. Results and Discussion

**Figure 1:**
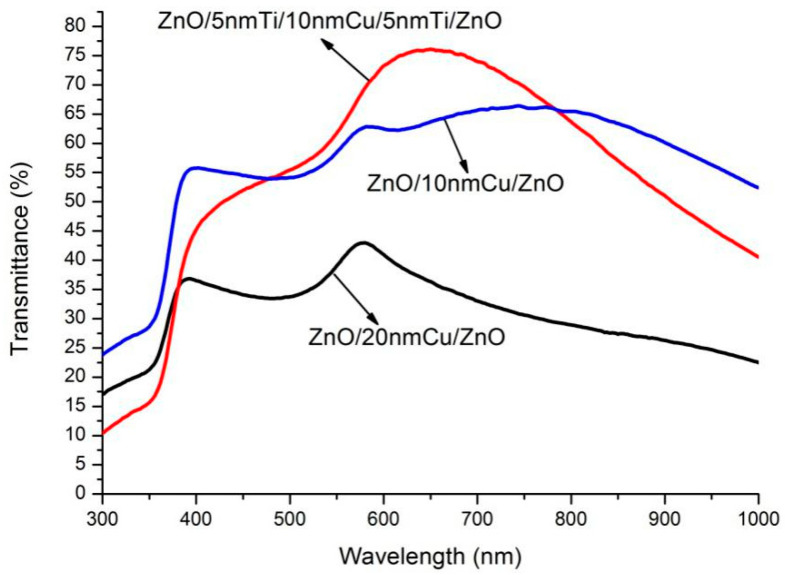
The optical transmission spectra of three unannealed ZnO transparent conductive thin-film systems.

The transmission spectra of unannealed ZnO/10nm Cu/ZnO, ZnO/20nm Cu/ZnO and ZnO/5nm Ti/10nm Cu/5nm Ti/ZnO, as measured by ultraviolet and visible spectrophotometer, are presented in Figure 1. It can be noted that the transmittance of ZnO/10nm Cu/ZnO is higher than that of ZnO/20nm Cu/ZnO. This is similar to previous experiment results showing that the transmittance of ZnO(Cu) thin-film systems decreases as Cu film thickness increases [10,16,17]. As listed in Table 1, the transmittance of ZnO/5nm Ti/10nm Cu/5nm Ti/ZnO is also higher than that of ZnO/10nm Cu/ZnO in the wavelength range of 470–780 nm. The reasons for this phenomenon need to be analyzed in combination with the characteristics of Cu and Ti. It has been proven that the reflective effect and light absorption of Cu are the two main influencing factors of transmittance reduction, and they have a different degree of influence at short and long wavelengths [10,22,23,24]. That is, the reflective effect of the Cu film layer is the main cause for the transmittance reduction in the short-wavelength region, while light absorption affects the long-wavelength region. To be specific, in short wavelengths (less than 470 nm), the extinction coefficients of Cu and Ti are close, which means that the introduction of Ti layers has little effect on the attenuation of light absorption. However, because Ti has a higher refractive index than Cu, the reflection effect is more obvious, which leads to the transmittance of ZnO/5nm Ti/10nm Cu/5nm Ti/ZnO thin film being lower than that of ZnO/10nm Cu/ZnO in this region. In the wavelength range of 470–780 nm, since the extinction coefficient of Ti is significantly lower than that of copper, the addition of the Ti layer is beneficial to transmittance by reducing the effect of light absorption, as shown in Table 1. It is worth mentioning that the transmittance of the sandwiched ZnO/Metal/ZnO film is lower than that of the ZnO single film with the same thickness due to the reflective effect and light absorption of the metal interlayer. This has been confirmed by previous study [16].

In order to further compare the electric conductivity of three thin films, their sheet resistances are exhibited in Table 2. ZnO/20nm Cu/ZnO thin film has the minimum sheet resistance, while ZnO/10nm Cu/ZnO has the maximum. The reason for the change of sheet resistance can be explained intuitively. As a semiconductor material, the ZnO thin film has a high resistivity in the ZnO/Metal/ZnO film structure, hence, the overall conductivity of the film structure depends largely on the sandwiched metal layer introduced to improve the conductivity. Therefore, the increase of the metal layer thickness greatly reduces the sheet resistances of the ZnO/Metal/ZnO multilayer film structure. On the other hand, because the conductivity of Cu is superior to that of Ti, the resistance of thin film with 20nm Cu is less than that of 5nm Ti/10nm Cu/5nm Ti, although the overall thicknesses of both of the metal layers is 20 nm.

It is expected that both optical transmittance and electric conductivity of transparent conductive films are maximized as they are two important parameters for transparent conductive thin film. In fact, optical transmittance and electric conductivity are two mutual constraints: improvement of optical transmittance (or electrical conduction) will lead to a decrease in electrical conduction (or optical transmittance). Therefore, an evaluation parameter named figure of merit was introduced by Haacke to evaluate the performance of transparent conductive film. The expression is defined as [25]:

(1) $$\Phi_{TC}=\frac{T^{10}}{R_{S}}$$

where *T* is the peak transmittance and *R_S_* is the sheet resistance. As listed in Table 2, the $\Phi_{TC}$ of ZnO/20nm Cu/ZnO is the minimum, which indicates that the overall performance of ZnO/20nm Cu/ZnO is the worst although it has the minimum sheet resistance. ZnO/5nm Ti/10nm Cu/5nm Ti/ZnO has the best performance.

It is known that the metal layer thickness has a significant effect on the performance of ZnO/Metal/ZnO transparent conductive thin films, which is also reflected in these films. Obviously, the figure of merit for ZnO/20nm Cu/ZnO is lower than that of ZnO/10nm Cu/ZnO. This indicates that the overall performance of ZnO/20nm Cu/ZnO is inferior to that of ZnO/10nm Cu/ZnO. The main reason is that, although an increase in thickness of the Cu layer is beneficial to the improvement of conductivity, it greatly reduces the transmittance. On the other hand, although the metal layer thickness of ZnO/5nm Ti/10nm Cu/5nm Ti/ZnO is also increased, its overall performance is improved, which is reflected in the increase of the figure of merit. It shows that the introduction of the Ti layer is helpful to the improvement of overall performance. This indicates that the Ti layer plays an important role in improving the overall performance of the conductive films.

**Figure 2:**
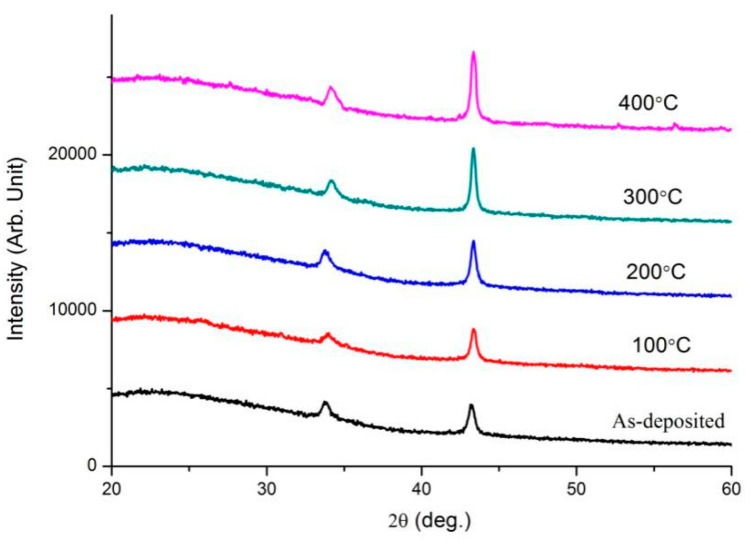
XRD spectra of ZnO(Cu) film system with varying annealing temperature.

**Figure 3:**
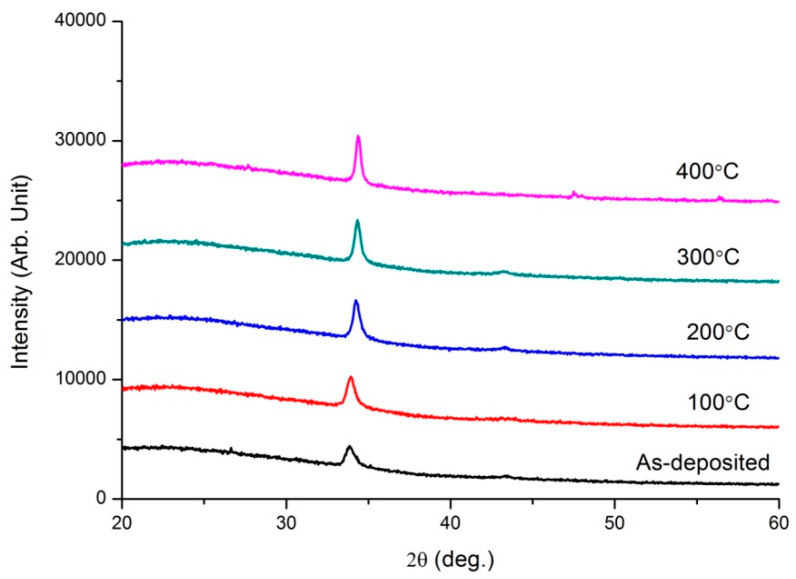
XRD spectra of ZnO(Ti/Cu) film system with varying annealing temperature.

To further study the performance evolution of ZnO transparent conductive thin film under various annealing conditions, two film systems, ZnO/20nm Cu/ZnO (abbreviated as ZnO(Cu)) and ZnO/5nm Ti/10nm Cu/5nm Ti/ZnO (abbreviated as ZnO(Ti/Cu)) were annealed at different temperature. Figure 2 and Figure 3 present the XRD spectra of the two film systems after annealing. As shown in Figure 2, each spectrum has two distinct diffraction peaks. The first peaks, belonging to ZnO, are hexagonal wurtzite, which indicates that ZnO films grow preferentially toward (002) orientation. The second peaks, corresponding to Cu(111), obviously indicate that Cu films grow toward (111) preferred orientation. The intensity of the Cu(111) peak increases with the temperature. This shows that annealing can improve the crystallization quality of the Cu layer. However, in Figure 3 it is observed that the ZnO hexagonal wurtzite polycrystalline structure with the (002) preferred orientation have been formed, and their peak intensities increase with the temperature. However, the diffraction peaks that can represent Cu are not obvious, which indicates that Cu does not crystallize very well. This is different from the XRD patterns of ZnO(Cu) thin films.

**Figure 4:**
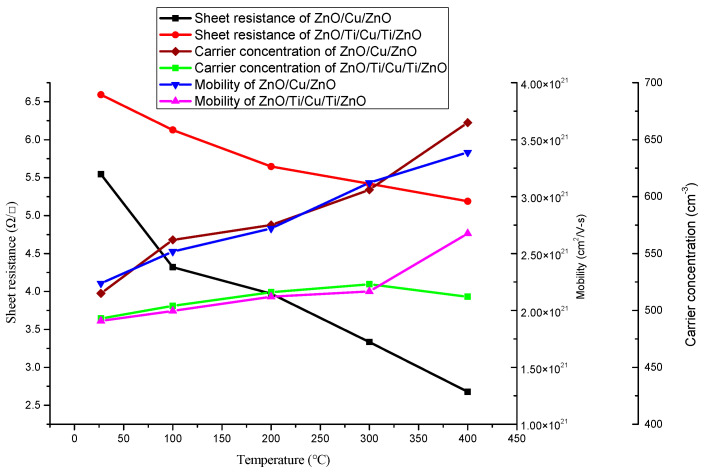
Electrical properties of two ZnO transparent conductive thin films with varying annealing temperatures.

The electrical properties of the two annealed thin-film systems are presented in Figure 4. The electrical conductivity of both film systems was improved with the increase of temperature. In detail, the sheet resistances of ZnO(Ti/Cu) thin films decreased from 6.59 Ω/sq; to 5.19 Ω/sq; when annealing temperature was raised from room temperature to 400 °C; the decrease of sheet resistance was smaller than that of ZnO(Cu) thin films, whose sheet resistance decreased from 5.54 Ω/sq; to 2.68 Ω/sq;. Their mobility shows an increasing trend with the temperature. The carrier concentration of ZnO(Cu) thin films tends to increase continuously while that of ZnO(Ti/Cu) thin films increases from room temperature to 300 °C and then decreases. The reasons for this change in electrical properties can be explained simply as follows. The annealing process is conducive to the improvement of the quality and the degree of crystallization of the thin film (as obviously shown in the XRD patterns in Figure 3). It leads to the decrease of free electron scattering, which induces the increase of the carrier concentration and mobility [26]. Further, for ZnO(Cu) thin films, the increase of the carrier concentration and mobility leads to the decrease of sheet resistance. On the other hand, for ZnO(Ti/Cu) thin films, the particle size of the nanomaterials will increase obviously as the annealing temperature goes up to 300 °C, after which the carrier concentration will finally decrease under the influence of the quantum size effect. If the rate of carrier concentration decrease is lower than that of mobility increase, the conductivity of the thin films is improved.

**Figure 5:**
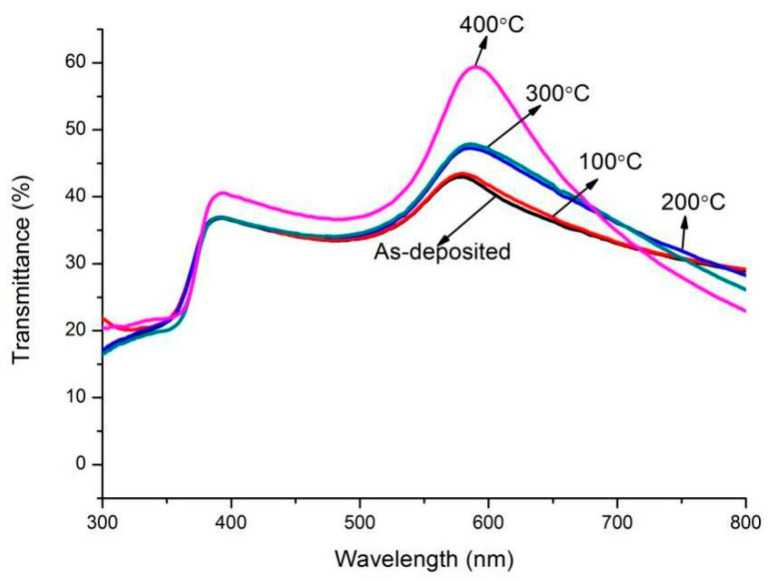
Optical transmission spectra of the ZnO(Cu) film system with varying annealing temperatures.

**Figure 6:**
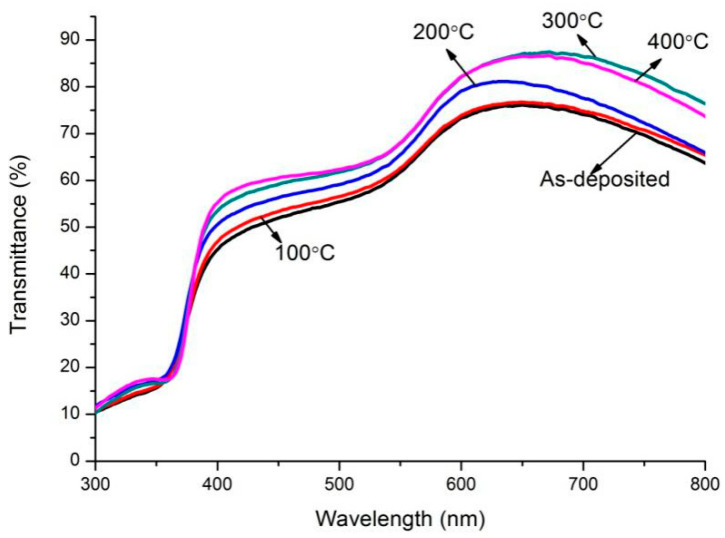
Optical transmission spectra of the ZnO(Ti/Cu) film system with varying annealing temperatures.

**Figure 7:**
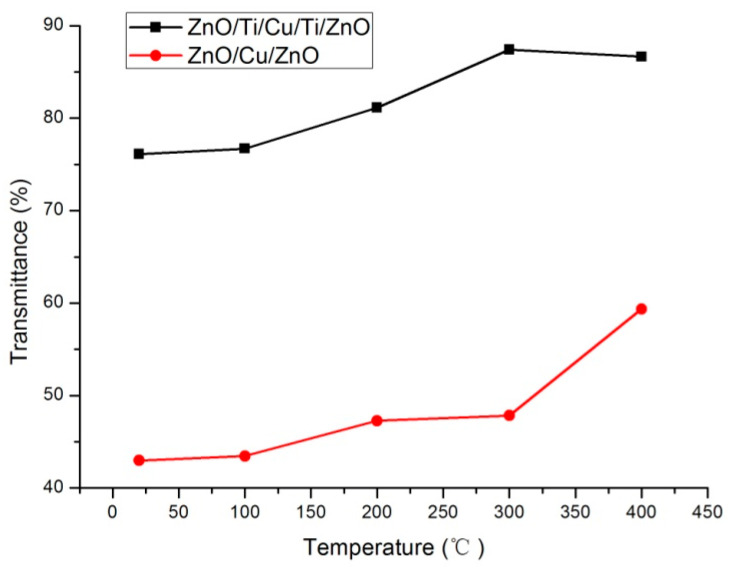
The maximum transmittance of the two multilayers with different annealing temperatures.

The transmission spectra of two annealed film systems are presented in Figure 5 and Figure 6. For the ZnO(Cu) film system, there are two crests in the spectra at wavelengths of about 380 nm and 580 nm. The wavelength shift of peak transmittance around the center wavelength of 380 nm is not obvious. However, the center wavelength of peak transmittance in long wavelengths shifts towards the longer wavelength regions. It can be noted that all the optical transmittances are lower than 60% in the range of wavelengths shown in the figure. It is also observed that there is only one crest in the spectrum, and all the peak optical transmittances are higher than 75% for ZnO(Ti/Cu) multilayers annealed at different temperatures. In order to clearly exhibit the relation between transmittance and temperature, the maximum transmittances of the two annealed multilayer film systems are presented in Figure 7. It is noted obviously that the transmittance of ZnO(Ti/Cu) thin films is higher than that of ZnO(Cu) thin films. In detail, the transmittance of ZnO(Cu) thin films increases with the annealing temperature. In particular, when the temperature increases from 300 °C to 400 °C, there is a large improvement in transmittance. But for ZnO(Ti/Cu) thin films, the transmittance shows a trend of increasing at first and then decreasing. That is, the multilayer has the maximum transmittance (about 87%) when the annealing temperature is 300 °C. Although annealing can improve the optical properties of the sandwiched ZnO/Metal/ZnO films to a certain extent, the reflective effect and light absorption of the metal layers still exist. Therefore, the average visible transmittance of sandwiched ZnO/Metal/ZnO films after annealing is lower than that of ZnO single film with the same thickness.

**Figure 8:**
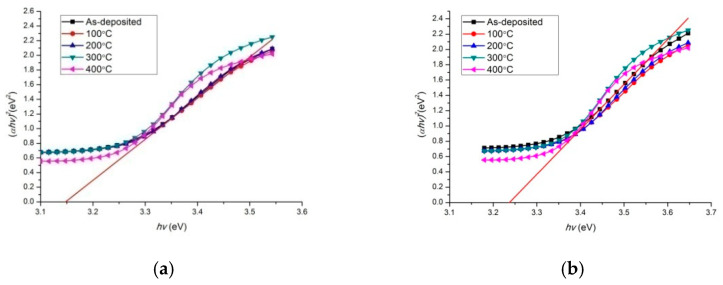
The relation curve between (*αhv*)^2^ and *hv* of two annealed film systems: (**a**) ZnO(Cu); (**b**) ZnO(Ti/Cu).

**Figure 9:**
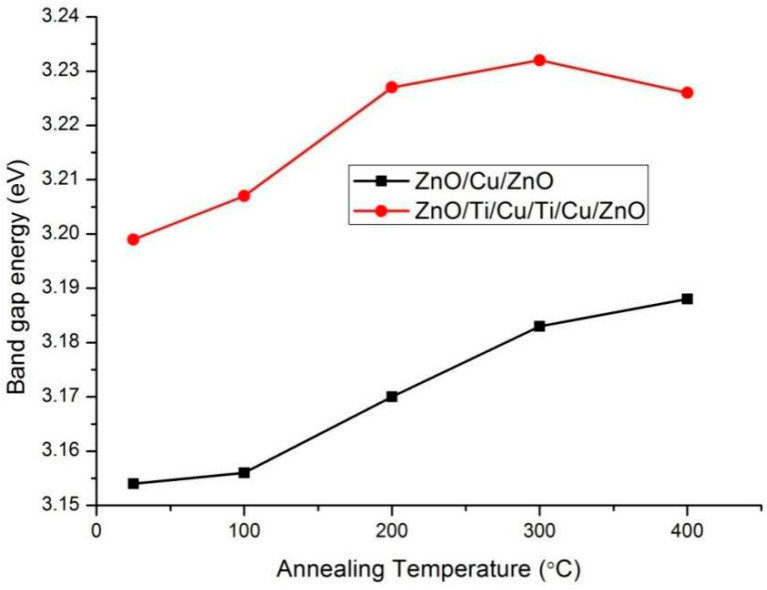
Bandgap energy of two annealed film systems.

According to the transmittance spectra in Figure 5 and Figure 6, the relationship curve between the (*αhv*)^2^ and *hv* of the two multilayer film systems with different annealing temperatures can be obtained, as shown in Figure 8, where *α* is the absorption coefficient and *h* is the photon energy. For ZnO/Metal/ZnO thin-film materials, the relationship between the *α* and *hv* can be expressed as [12,18,27,28,29,30]:

(*αhv*)^2^ = Λ(*hv* − *E_g_*)

where Λ is a constant and *E_g_* is the optical bandgap. Normally, *E_g_* is extrapolated from the linear part of the relationship curve to the *hv* axis, as shown in Figure 8. The optical bandgaps of two annealed film systems are presented in Figure 9. The optical bandgap of ZnO obtained from ZnO(Cu) thin films (3.15~3.19 eV) is lower than that of ZnO(Ti/Cu): 3.20~3.23 eV, which varies near the theoretical value (~3.2 eV) [31,32]. In particular, the bandgap of ZnO(Cu) thin film increases with temperature, while that of ZnO(Ti/Cu) first increases and then decreases. The increase of bandgap after annealing is caused by the Burstein–Moss migration effect [33], which is related to the increase of carrier concentration in the film (Figure 4). The increased carriers fill in the lower energy level of the conduction band, making the valance electrons transfer to the higher energy level, thus increasing the bandgap width. As to the ZnO(Ti/Cu) thin films, the decrease of the bandgap between 300 °C and 400 °C is due to the increase of the particle size of the film, whose quantum size effect reduces the carrier concentration, leading to the bandgap narrowing.

**Figure 10:**
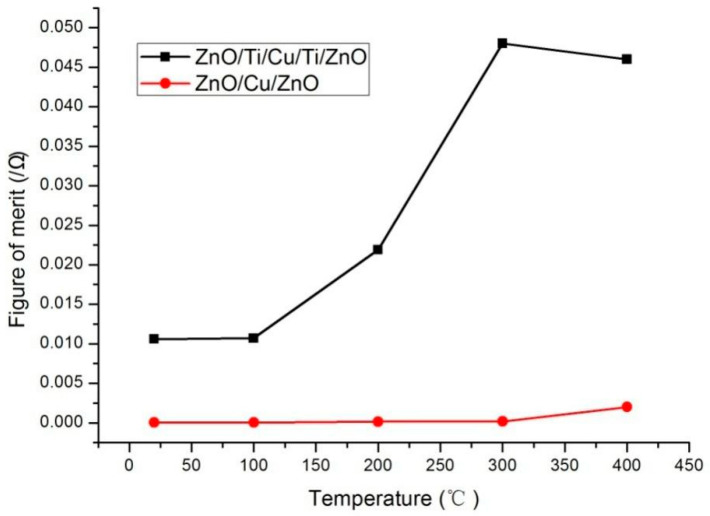
The figures of merit of the ZnO(Cu) and ZnO(Ti/Cu) thin films with varying annealing temperatures.

The figures of merit of the ZnO(Cu) and ZnO(Ti/Cu) film systems after annealing are presented in Figure 10. The figure of merit of ZnO(Ti/Cu) is higher than that of ZnO(Cu). This shows that the performance of ZnO(Ti/Cu) is superior to that of ZnO(Cu). To be more specific, the higher annealing temperature increases the figure of merit for ZnO(Ti/Cu), but not obviously for ZnO(Cu). In other words, annealing has greatly improved the performance of ZnO(Ti/Cu). The figure of merit is highest when the annealing temperature is 300 °C although its conductivity is worse than thin film annealed at 400 °C. This means that the ZnO(Ti/Cu) thin film annealed at 300 °C has the best overall performance [17].

## 4. Conclusions

Two metal-sandwiched ZnO transparent conductive thin-film systems (50nm ZnO/Cu/50nm ZnO and 50nm ZnO/Ti/Cu/Ti/50nm ZnO) were fabricated, and the comparative analysis of their properties shows that the introduction of Ti layers can improve the overall performance of the film while maintaining the same overall metal layer thickness. The influence of the annealing temperature on the performance of two film systems was studied. The XRD patterns show that ZnO films have the (002) preferred orientation in both film systems, and the Cu films have [111] crystal orientation in 50nm ZnO/Cu/50nm ZnO films but do not crystallize very well in 50nm ZnO/Ti/Cu/Ti/50nm ZnO films. Annealing improved the conductivity of the two films due to a combination of changes in carrier concentration and mobility (although carrier concentration did not always increase with annealing temperature). The optical transmittance of ZnO(Ti/Cu) thin films with different annealing temperatures were higher than that of ZnO(Cu) thin films. The transmittance of ZnO(Cu) thin films increased with the annealing temperature, while that of ZnO(Ti/Cu) thin films increased from 100 °C to 300 °C and then decreased with higher temperatures. The bandgap of ZnO obtained from ZnO(Cu) thin films increased with temperature but remained lower than that of ZnO(Ti/Cu), which first increased and then decreased with temperature. The figure of merit of the ZnO(Ti/Cu) thin film is higher than that of ZnO(Cu), which indicates that the performance of ZnO(Ti/Cu) thin films is superior to ZnO(Cu). By comparing figures of merit, it can be determined that annealing can improve the performance of the film systems.

## Figures and Tables

**Table 1:** The refractive index and extinction coefficient of ZnO, Cu and Ti at different wavelengths [24].

Wavelength (nm)	Refractive Index			Extinction Coefficient	
	ZnO	Cu	Ti	Cu	Ti
387.44	2.264	1.231	1.500	2.068	2.12
413.27	2.181	1.185	1.590	2.208	2.17
442.79	2.122	1.168	1.680	2.363	2.25
476.85	2.078	1.152	1.750	2.504	2.34
516.58	2.045	1.12	1.810	2.603	2.47
539.04	2.032	1.038	1.860	2.592	2.56
563.55	2.019	0.826	1.920	2.602	2.67
590.38	-	0.468	2.010	2.809	2.77
619.9	-	0.272	2.110	3.326	2.88
652.53	-	0.214	2.220	3.667	2.99
688.78	-	0.213	2.360	4.043	3.11
729.294	-	0.223	2.540	4.433	3.23
826.53	-	0.26	2.980	5.26	3.32

**Table 2:** Sheet resistance and figure of merit of the three ZnO transparent conductive thin films.

Figure	Sheet Resistance (Ω/sq;)	Figure of Merit
ZnO/10nm Cu/ZnO	10.1	0.00181
ZnO/20nm Cu/ZnO	5.5	0.00004
ZnO/5nm Ti/10nm Cu/5nm Ti/ZnO	6.6	0.01060

## Data Availability

Not applicable.
